# Association of microbial community structure with gill disease in marine-stage farmed Atlantic salmon (*Salmo salar*); a yearlong study

**DOI:** 10.1186/s12917-024-04125-5

**Published:** 2024-08-01

**Authors:** Morag Clinton, Adam J. Wyness, Samuel A. M. Martin, Andrew S. Brierley, David E. K. Ferrier

**Affiliations:** 1 Scottish Oceans Institute, University of St Andrews, St Andrews, UK; 2 Department of Veterinary Medicine, University of Alaska Fairbanks, Fairbanks, AK USA; 3 Scottish Association for Marine Science, Oban, UK; 4 Scottish Fish Immunology Research Centre, University of Aberdeen, Aberdeen, UK; 5 Sitka Sound Science Center, Alaska Sitka, USA

**Keywords:** Fish health, Gill microbiome, Atlantic salmon aquaculture, Gill pathology, Pathobiome, Dysbiosis

## Abstract

**Background:**

Understanding the relationship between resident microbiota and disease in cultured fish represents an important and emerging area of study. Marine gill disorders in particular are considered an important challenge to Atlantic salmon (*Salmo salar*) aquaculture, however relatively little is known regarding the role resident gill microbiota might play in providing protection from or potentiating different gill diseases. Here, 16S rRNA sequencing was used to examine the gill microbiome alongside fish health screening in farmed Atlantic salmon. Results were used to explore the relationship between microbial communities and gill disease.

**Results:**

Microbial community restructuring was observed throughout the sampling period and linked to varied drivers of change, including environmental conditions and severity of gill pathology. Taxa with significantly greater relative abundance on healthier gills included isolates within genus *Shewanella,* and taxa within family *Procabacteriaceae*. In contrast, altered abundance of Candidatus *Branchiomonas* and *Rubritalea* spp. were associated with damaged gills. Interestingly, more general changes in community richness and diversity were not associated with altered gill health, and thus not apparently deleterious to fish. Gross and histological gill scoring demonstrated seasonal shifts in gill pathology, with increased severity of gill damage in autumn. Specific infectious causes that contributed to observed pathology within the population included the gill disorder amoebic gill disease (AGD), however due to the uncontrolled nature of this study and likely mixed contribution of various causes of gill disease to observed pathology results do not strongly support an association between the microbial community and specific infectious or non-infectious drivers of gill pathology.

**Conclusions:**

Results suggest that the microbial community of farmed Atlantic salmon gills undergo continual restructuring in the marine environment, with mixed influences upon this change including environmental, host, and pathogenic factors. A significant association of specific taxa with different gill health states suggests these taxa might make meaningful indicators of gill health. Further research with more frequent sampling and deliberate manipulation of gills would provide important advancement of knowledge in this area. Overall, although much is still to be learnt regarding what constitutes a healthy or maladapted gill microbial community, the results of this study provide clear advancement of the field, providing new insight into the microbial community structure of gills during an annual production cycle of marine-stage farmed Atlantic salmon.

**Supplementary Information:**

The online version contains supplementary material available at 10.1186/s12917-024-04125-5.

## Introduction

Fish gills are exposed to the surrounding environment and are therefore a site for surface proliferation and ingress of pathogens leading to disease [1, 2]. Gill diseases are a particular challenge to aquaculture of Atlantic salmon (*Salmo salar*), an industry where gill disorders are considered a leading cause of production loss in the marine stage of production [3]. Epitheliocystis and Tenacibaculosis are both gill diseases caused by bacteria in the marine environment that negatively impact Atlantic salmon [4–6]. However, emerging research in the field of animal health suggests that the bacteria of fish tissues are not only associated with disease occurrence but may also play an important role in host disease resistance and even immunological defense [7, 8]. Resident microbial communities are suggested to have a function as part of the mucosal barrier defense system, helping to protect against pathogen invasion and disease [9, 10]. Thus, an understanding of the microbial community of gills may be highly relevant to preventing or mitigating gill disease. Manipulation of resident microbial communities is emerging as a critical area of study to optimize fish health [11, 12], with many stakeholders interested in what constitutes an advantageous microbial community, as well as what represents a disrupted microbial community [13, 14].

Existing research in salmonids demonstrates that the communities of bacteria resident on the gills and skin are influenced by many factors. Environmental factors such as water temperature, salinity, and pH have all been demonstrated to impact resident microbiomes of these environmentally-facing organs [15–17]. However, despite this, studies show that the microbiota of gills and skin of fish like Atlantic salmon remain distinct from surrounding environmental communities [18, 19]. This may be due in part to the fact that, in addition to environmental influences, host factors also dictate resident microbial community structure. Whilst microbiota acquired from the environment can be successful or unsuccessful in their colonization of tissue by random change [20], current hypotheses in the field of Atlantic salmon microbiology and immunology also suggest a role of the host immune system in modulation of resident microbiota not only in pathogen removal but also in allowing the proliferation of beneficial microorganisms [21, 22]. Disruption of the resident microbiome in fish may therefore result in negative host outcomes and has been shown to allow pathogen proliferation as well as increased susceptibility to disease [23]. The presence of disease itself can significantly alter microbial community structure. Parasitism of Atlantic salmon by sea lice (*Lepeophtheirus salmonis*) has been described as perturbing the microbiome [24], and infection by microbial pathogens *Photobacterium damselae* and *Tenacibaculum maritimum* have been shown to significantly alter fish skin microbial consortia during disease outbreaks [25, 26]. Emerging research demonstrates that bacteria act as an important component of various mixed and multifactorial gill pathologies in Atlantic salmon, including Complex Gill Disease (CGD) and Amoebic Gill Disease (AGD) [27–29], both important infectious conditions in Atlantic salmon aquaculture. During AGD, gills become colonized by the protozoan parasite *Neoparamoeba perurans*, with recent research demonstrating that microbial communities of Atlantic salmon gills are also altered during AGD disease events. Some authors therefore suggest a role of the microbiota in gill health outcomes with AGD [31–34]. Identification of high prevalence or altered abundance of specific microorganisms in conjunction with gill disease might therefore be considered an indication of an unhealthy microbial community, whether as a cause of or as a symptom of disease.

Overall, in addition to the challenges of bacterial overgrowth and infections to fish health [3], microbial interactions between the host and resident microbial communities are suggested to be of importance in determining disease outcomes of other infectious conditions. However, currently relatively little is known regarding the role resident gill microbiota might play in providing protection from or potentially acting as a predisposing factor to gill diseases. Whilst it is challenging to link negative fish health outcomes to altered microbial community structure directly, microbial consortia generally are clearly of importance in the complex aetiology of various Atlantic salmon gill diseases. This study sought to explore the resident microbiota of Atlantic salmon gills with a focus on identifying any association of the resident community with negative health consequences in fish. Our aim was to investigate the microbial community during an annual marine production cycle in a commercial setting to identify any link between gill community restructuring and gill disease. To achieve this aim, the gill community was sampled from Atlantic salmon alongside gill health monitoring, both visually and via histopathology. Results were subsequently explored to determine any association of the microbial community with negative health outcomes in fish.

## Materials and methods

Fish were sampled from a population of farmed Atlantic salmon on the West Coast of Scotland (UK) with samples collected to assess the microbial community and gill health status of fish. Fish were maintained as part of a single cohort that altered in age during the study, starting in May 2017 as approximately 1 year old smolts recently transferred to seawater (average weight 85.5 g ± 17.3 g) through to pre-harvest fish (average weight 3550.0 g ± 1035.1 g) the following June. Fish were of identical genetic background, stocked from a single source. Sampling was initiated approximately two weeks following transfer from freshwater. Fish were maintained as a single population within a marine cage as part of stock in an Atlantic salmon commercial aquaculture facility. During the 13-month duration of the project, fish were subject to various husbandry management interventions by on-farm personnel. These included introduction of lumpfish (*Cyclopterus lumpus* L. 1758) and Ballan wrasse (*Labrus bergylta*) to the marine cage, as well as treatment of fish with both hydrogen peroxide and warm water for control of sea lice (*Lepeophtheirus salmonis*). The timing of these events were detailed in Table S1.

Individual fish were obtained by food incentivized crowd-netting from a single pen of the Scottish Sea Farms (SSF) facility. Hand-netted fish were euthanized immediately by farm staff using immersion in MS222 to facilitate sample collection and prevent disruption of gill tissues [35, 36]. This methodology was approved by the Animal Welfare and Ethics Committee at the University of St Andrews in line with European Union directive 2010/63UE. Samples were collected at 11 time points throughout the annual marine production cycle. These time points were labelled by date and grouped based on relative timing in the year (Table S1). The timing of sampling was variable to coincide with relevant on-farm activities and to avoid husbandry staff workload clashes. A total of 12 individuals were obtained at each sampling visit for a final total of 132 fish sampled over a period of 13 months.

### Gross gill assessment

Assessment of clinical gill pathology was performed through gross scoring of lesions on gills before sample collection. Gross scoring was conducted as described in Table 1, a methodology modified from commonly performed assessments by the producer and published scoring systems [37]. Gross assessments and scoring assessments were conducted consistently by a single experienced observer. Gill samples for microbial community analysis were sampled as previously described [38]. Briefly, this involved excising the first left gill arch and placing a portion of tissue (described from here on as “biopsies”) in RNAlater solution. This approach was designed to maximize inclusion of any pathogenic bacteria in the obtained sample [38]. To maintain as sterile a sampling environment as possible, following capture, fish were immediately moved to a controlled environment and sampled upon food-grade aluminum foil [39]. Sterile instruments and blades were used and changed between each fish. The portion of gill collected for each biopsy was taken consistently from the top of the first left gill arch regardless of location of observable pathology. Gills were not washed or dried prior to placement in fixative to avoid disruption of the mucus layer and its associated microbiome [38]. Biopsies were fixed in 25 ml RNAlater solution (ThermoFisher Scientific) and maintained at ambient temperature for approximately 24 h before cold storage at -20 °C on return to the laboratory. The second left gill arch was excised and fixed in its entirety in 10% neutral buffered formalin. Formalin fixed material was maintained at ambient temperature until processing for histology.

**Table 1:** Gross scoring system for visual (macroscopic) assessment of gill pathology

Gross classification	Gross lesions associated with AGD	Generalized gill damage
None (0)	None – no mucoid plaques observed	Gills appear healthy, with no discoloration or visible lesions, and with full length filaments
Very slight (1)	Presence of white/grey mucoid plaques in low numbers (1–2) on a single gill arch.	Shortening and/or discoloration of filaments (<5% surface area)
Localized (2)	Presence of white/grey mucoid plaques in low numbers (1–2) on multiple gill arches (> 1)	Shortening and/or discoloration of filaments (5–20% surface area single gill arch)
Multifocal (3)	Presence of white/grey mucoid plaques (3–5) on multiple gill arches (> 1)	Shortening and/or discoloration of filaments (5–20% of surface area multiple gill arches)
Extensive (4)	Presence of white/grey mucoid plaques (> 5) on multiple gill arches	Shortening and/or discoloration of filaments (> 20% of surface area multiple gill arches)

Gross scoring system for visual inspection of Atlantic salmon gills. All gills were visually assessed, back and front, by a single observer to identify and determine the extent of any pathology. Examples of gross lesions associated with AGD and generalized gill damage are included within supplementary materials (Figure S1)

### Histopathology

Formalin-fixed gill samples were commercially processed and stained by the Fish Vet Group, Inverness, UK to produce hematoxylin and eosin (H&E) stained gill section slides. Histological slides obtained from the Fish Vet Group were then read blind and in random order. Slides were assessed and imaged by a single author using a Nikon Ni-U microscope for detailed qualitative pathological assessment, with each gill section examined for changes suggestive of gill disease. Semi-quantitative scoring was then performed by the same assessor using a previously described composite scoring system by Mitchell et al. [40], modified slightly for the needs of this research (Table S2). This allowed generation of an overall composite score using the existing scoring system’s listed primary and secondary criteria. A 0 - 3 scale was used for each index (primary) criterion alongside a 0 - 1 scale for ancillary (secondary) criteria. Scores were then combined to create a total score for each fish as a quantitative assessment of gill pathology [40]. Modifications to the scoring system included consideration of discrete scores from individual index (primary) and ancillary (secondary) criteria to obtain Additional quantitative information regarding the pathology contributing to cumulative gill scores. Index criteria were cellular hyperplasia, lamellar fusion, cellular abnormalities, and lamellar oedema. These index criteria were individually graded from 0 - 3. Ancillary (secondary) criteria for specific disease-causing organisms and other changes were described as present or unobserved. Use of the combined scores in line with the published scoring system provided an indication of general gill damage in each fish. This was in addition to detailed qualitative pathological assessment. Care was taken to ensure accuracy of histological interpretation, particularly when the presence of artefacts was detected. Based on the overall scores assigned using this protocol, gill histopathology for each fish was classified as having either none (0 - 3), mild (4 - 6), moderate (7 - 9) or severe (10 and over) change. None and mild changes (scored 0 - 6) were considered overall to be a presentation consistent with relatively healthy gills, where pathology was of minor clinical significance. Gill sections classified as having moderate or severe pathology (with a composite score over 6) were considered to be relatively unhealthy, with pathology of clinical significance. These classifications are in line with previous use of the published scoring system [40].

### DNA extraction and quality control

DNA was extracted from RNAlater fixed gill samples using a modified protocol for the DNeasy Blood and Tissue extraction kit (Qiagen) with a phenol–chloroform step as previously described [38]. Briefly, gills were mechanically disrupted before the addition of tissue digestion reagents for overnight incubation. Following digestion, a phenol–chloroform extraction step was performed to remove excess protein [41], followed by completion of the Dneasy Blood & Tissue kit protocol according to the manufacturer’s guidelines. DNA quality and quantity were analyzed using a Nanodrop 1000 spectrophotometer. Extractions of any sample lower than 80 ng/μl were repeated from reserved gill biopsy material. Purity and integrity of DNA was checked by measuring absorbance at 260:280 nm (> 1.8) and 230:260 nm (> 1.8) using a nanodrop, as well as through use of a high molecular weight DNA smear in 1% agarose gels stained with ethidium bromide. Duplicate DNA extractions were performed and pooled to a final DNA concentration of 45 ng/μl for each fish.

### 16S rRNA sequencing

Amplicon generation and library preparation for high-throughput sequencing were performed in-house and largely in accordance with the Illumina Metagenomic sequencing library preparation protocol (Illumina, 2013). Amplification using primers targeting the V3-V4 region of 16S rRNA was conducted as previously described [38], with DNA concentrations of extractions, stocks, and cleaned PCR reactions obtained through use of the Qubit dsDNA BR Assay and Qubit dsDNA HS Assay kits (ThermoFisher Scientific) using Qubit 4.0 Fluorometer (Invitrogen, ThermoFisher Scientific) according to the manufacturer’s instructions. Products were pooled in equimolar concentrations for a final library concentration of 4 nM. The resultant library was then denatured and hybridized according to the manufacturer’s recommendations with a 20% PhiX spike-in [38]. Pooled tagged amplicons were sequenced in a single run using the 2 × 300 bp MiSeq reagent kit v3 (Illumina) according to the manufacturer’s protocol.

### Metagenomics workflow

Demultiplexed next generation data from the sequencing of prepared libraries was denoised and filtered using open source DADA2 [42] within Qiime2 v2019.2 [43, 44]. The following parameters were used for DADA2; trunc_len_f: 300; trunc_len_r: 279; trim_left_f: 27; trim_left_r: 15; max_ee: 2; trunc_q: 2; chimera_method: consensus; min_fold_parent_over_abundance: 1, to produce a table of counts of reads of amplicon sequence variants (ASVs) for each sample [45, 46]. Taxonomy was assigned to results using a naïve bayes classifier [47] within QIIME2 with default parameters and the SILVA 138.1 SSU non-redundant reference database [48]. Additional confirmation of taxonomy was performed for high prevalence ASVs (> 5% abundance) by uploading sequences for comparison against NCBI Standard databases in the online ‘BlastN suite’ online tool using default parameters. Amplicons were re-classified to genus level or higher using BlastN based off a percent identity score of ≥ 97% in instances where disagreement was observed between the SILVA 138.1 and NCBI reference databases. Sequences assigned to chloroplasts, archaea, mitochondria and reads unassigned below kingdom level were removed for generation of the final dataset. Abundance profiles were calculated based on total read counts in individual samples for assessment of beta diversity. Alpha-diversity metrics were calculated from treatment medians of a rarefied dataset (1200 reads) where only isolates that reached the rarefaction curve plateau were included (Figure S2). Resemblance matrices, beta diversity metrics and multivariate analysis were performed using the programs Primer version 7 and Permanova + [49, 50]. Additional figure generation and statistical testing was performed using Vegan and Bioconductor packages in R 3.5.0 [51]. Figures were generated using Primer version 7 and R 3.5.0. Additional statistical testing was performed using in-built features of R.

## Results

### Gross pathology

**Fig. 1:**
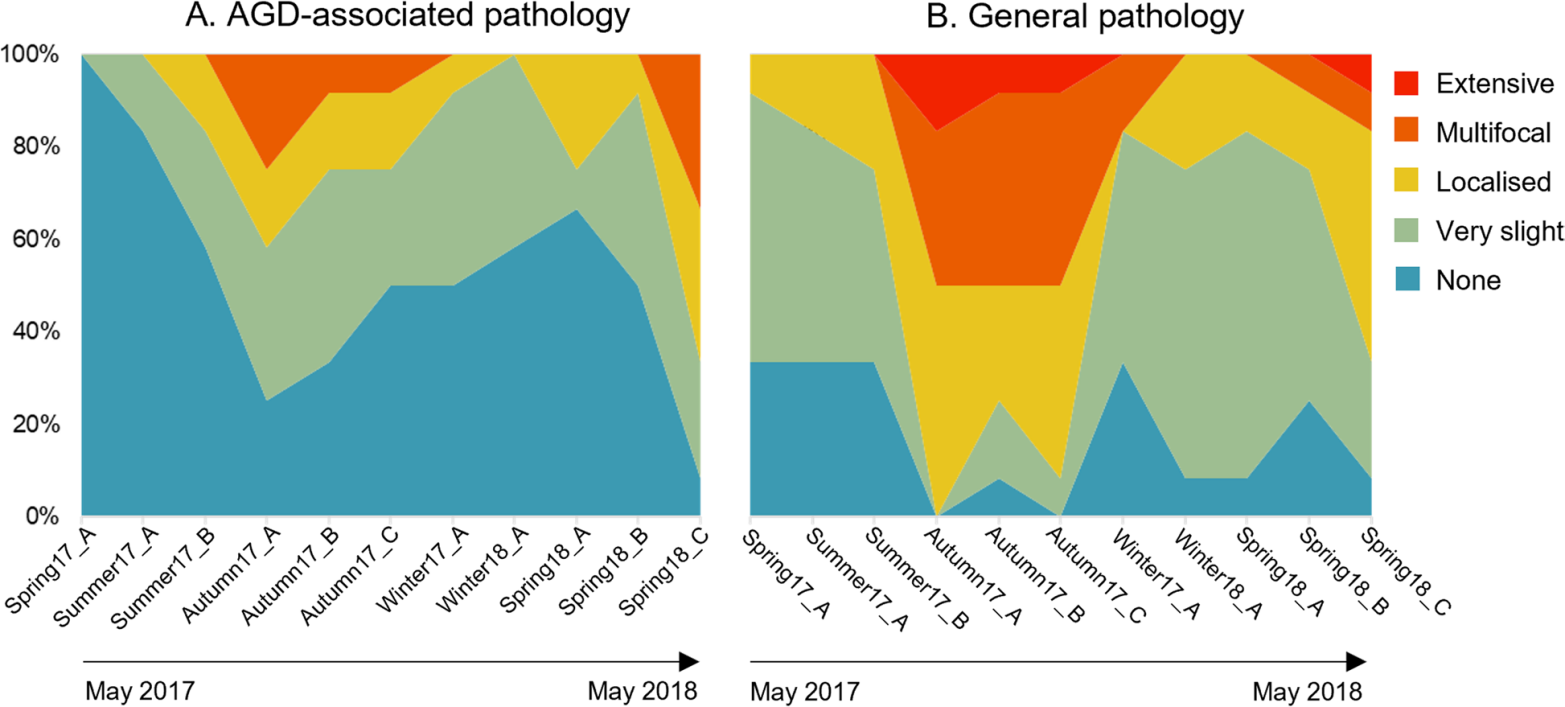
Visual scoring of macroscopic (gross) damage to gills. Results of visual scoring of macroscopic (gross) damage to gills during each site visit (total 12 fish per visit). Use of gross scoring criteria (Table 1) allowed numerical classification of gross lesions associated with AGD and more general gill pathology for each fish. For each sampling visit (start of May 2017 to end of May 2018) AGD-associated (**A**) and general pathology (**B**) scores are illustrated

Assessment of macroscopic (gross) pathology of gill tissues gave an indication of the overall level of damage in gills. For each fish all gill arches both left and right were visualized and an overall score assigned according to the presence or absence of characteristic gill pathology (Table 1). Visible pathology was considered either to be suggestive of AGD (with characteristic grey/white mucoid plaques) or more generic gill pathology (Figure S1). Results from this gross scoring demonstrate a seasonal trend in severity of macroscopic change within the sampled population. During early sampling, following entry to the marine environment, gills were not observed to have mucoid plaques associated with AGD. Mucoid plaques considered characteristic of AGD were first observed in the population during the summer. Highest scores for both mucoid plaques associated with AGD and more generalized gross change were observed during sampling visits coinciding with late summer into autumn and early winter (Fig. 1).

### Histopathology

**Fig. 2:**
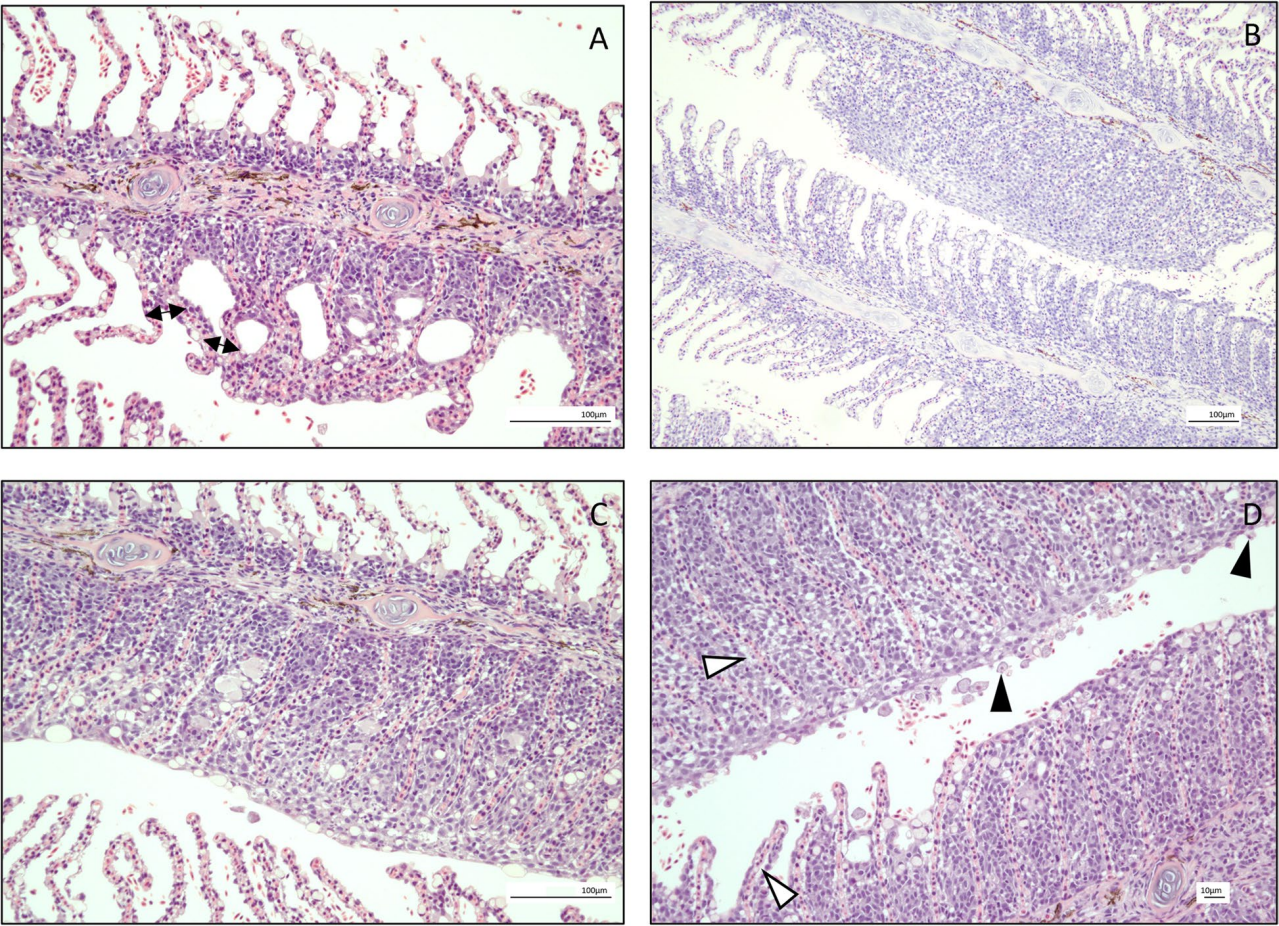
Examples of histopathology from the sampled population. Histological sections of H&E stained gills. **A** Gill section with focal lamellar fusion (double-headed arrows), mild epithelial hyperplasia, and a small number of individual hypertrophic cells. The entire section was overall scored as 1/3 for lamellar fusion, 1/3 for cellular hyperplasia, and 1/3 for cellular abnormalities. **B** Gill section with multifocal lamellar fusion and epithelial hyperplasia. The entire section was scored overall as 2/3 for lamellar fusion, 2/3 for cellular hyperplasia, and 1/3 for cellular abnormalities. **C** Gill section with multifocal lamellar fusion, epithelial hyperplasia, and foci of goblet cell hyperplasia alongside cell death. The entire section was scored overall as 2/3 for lamellar fusion, 2/3 for cellular hyperplasia, and 1/3 for cellular abnormalities. **D** Gill section with diffuse lamellar fusion and epithelial hyperplasia, presence of mild oedematous change (white arrowheads), and with noted presence of amoebic organisms (black arrowheads). The entire section was scored overall as 3/3 for lamellar fusion, 3/3 for cellular hyperplasia, 1/3 for cellular abnormalities, and 1/3 for lamellar oedema, with noted amoeba presence

Sampled fish demonstrated varying degrees of microscopic gill pathology both between and within sampling groups. Index (primary) criteria of lamellar fusion, cellular abnormalities, cellular hyperplasia, and oedema were observed throughout the population. Commonly observed pathological features included cellular hyperplasia, prominently of epithelial cells, and lamellar fusion (Fig. 2). A total of 112 sampled fish were identified to have some degree of cellular hyperplasia, 69 fish were noted to have lamellar fusion, 63 fish had cellular abnormalities, and 19 fish demonstrated oedema. Regarding ancillary (secondary) criteria, 46 fish demonstrated evidence of inflammatory infiltration, 55 fish had evidence of circulatory disturbances, and 50 fish were noted to have cellular hypertrophy (Table S3). In addition to non-specific gill pathology, specific pathogenic organisms were also observed in a proportion of gill sections. This included observation of amoebic organisms with an internal parasome consistent in appearance with *Neoparamoeba* in the gill tissue of 10 individuals [52]. No evidence of bacterial overgrowth or colonization was directly observed on the surface of gill sections, and the presence of epitheliocystis-type lesions were not a common feature within the population. No gill pathology considered characteristic of Salmon Gill Pox virus or microsporidian *Desmozoon lepeophtherii* present was noted [53, 54]. Based on existing literature, fish can be considered to have been suffering from the infectious condition AGD when amoebic organisms with a parasome and hyperplastic gill changes are concurrently observed in gill sections [37, 55–58]. A total of 10 individuals met this case definition of AGD, making AGD the most commonly observed specific infectious agent of gill disease in the sampled population.

**Fig. 3:**
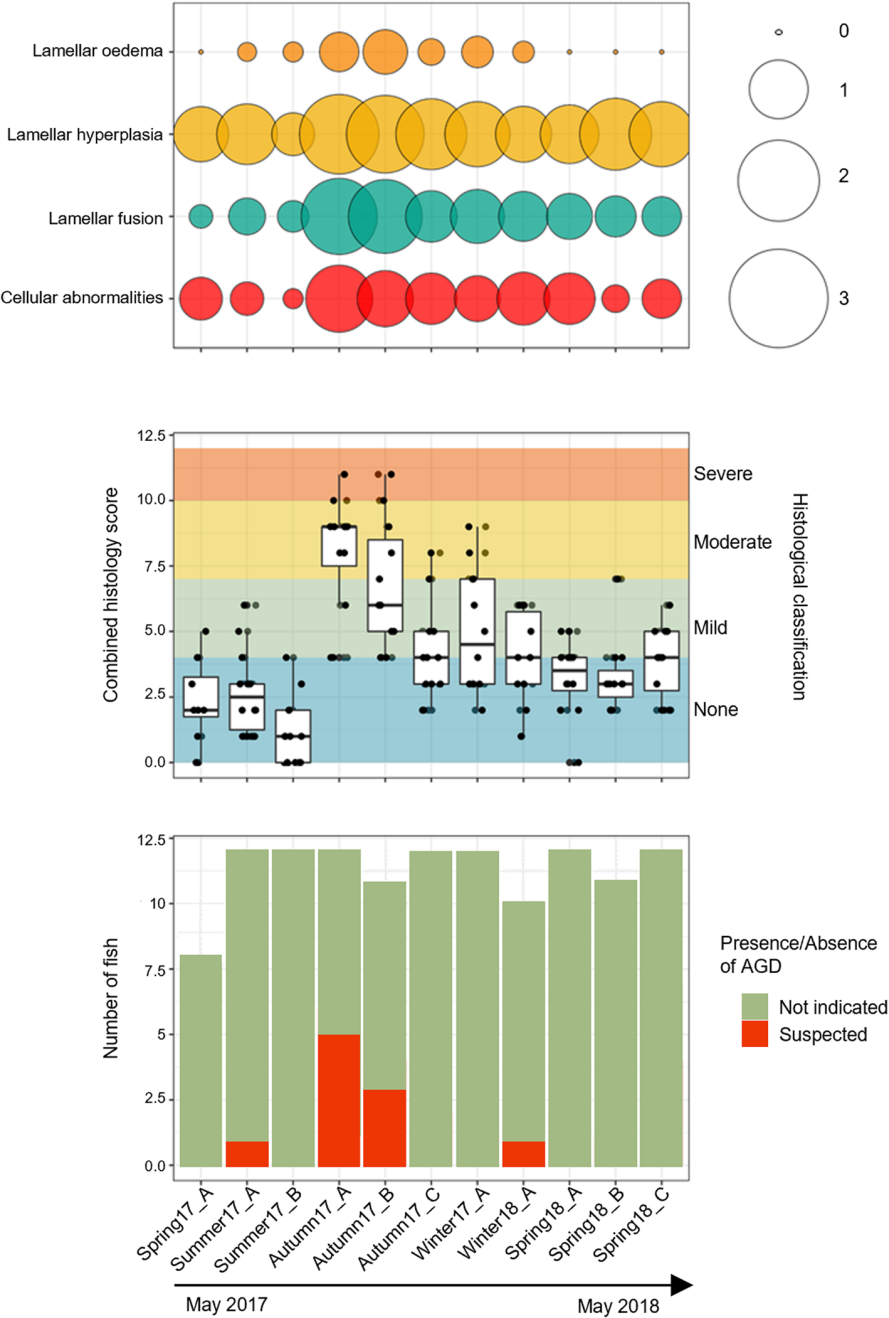
Results of scoring of microscopic damage to gills. Sampling visits from May 2017 to May 2018 represent the x axis of all figures. Bubble graph (top): Average histological scores assigned to index (primary) criteria across the sampling timeline are illustrated, where larger circles denote a higher average score at each sampling point. Box plot (middle): Overall histological scores from for each sampled fish, with background colors denoting how fish gills were classified based on their composite scores (none, mild, moderate, or with severe histological change). Points represent individual fish. Data for Top and Middle charts can be found in Table S3. Stacked bar chart (bottom): Total numbers of fish for which histological assessment of gills was performed are plotted. Those fish where histological sections were not considered diagnostic due to insufficient artifact free tissue have been removed from the analysis. Colors differentiate sampled fish as those meeting the case definition of AGD infection (red) and those where evidence was not present or insufficient for diagnosis of AGD (green)

Use of the adapted semi-quantitative histology scoring system allowed numerical classification of gills, with total gill scores ranging from 0 to 11 in the sampled population. These values were obtained through combining scores from index (key) and ancillary (secondary) criteria [40]. The breakdown of scoring results for index and ancillary criteria are presented in Table S3. Seasonal trends of index (primary) criteria graded 0 - 3 for each fish were apparent within the dataset (Fig. 3). Highest average index criteria scores were seen in fish sampled Autumn 2017. Similar trends are visible in cumulative score values, with notable increase in histology scores between Summer17_B (July 2017) and Autumn17_A (September 2017) sampling visits. These trends correlate temporally with trends seen in gross gill pathology scores, where more severe gill pathology appears to occur in fish sampled through autumn (Fig. 1). When fish are divided based on those meeting the histological case definition for AGD infection and those that did not, the majority of AGD cases are also observed during the sampling visits of Autumn17_A though _C, again mirroring trends in gross score severity (Fig. 3).

### 16S rRNA sequencing results

#### Community results

**Fig. 4:**
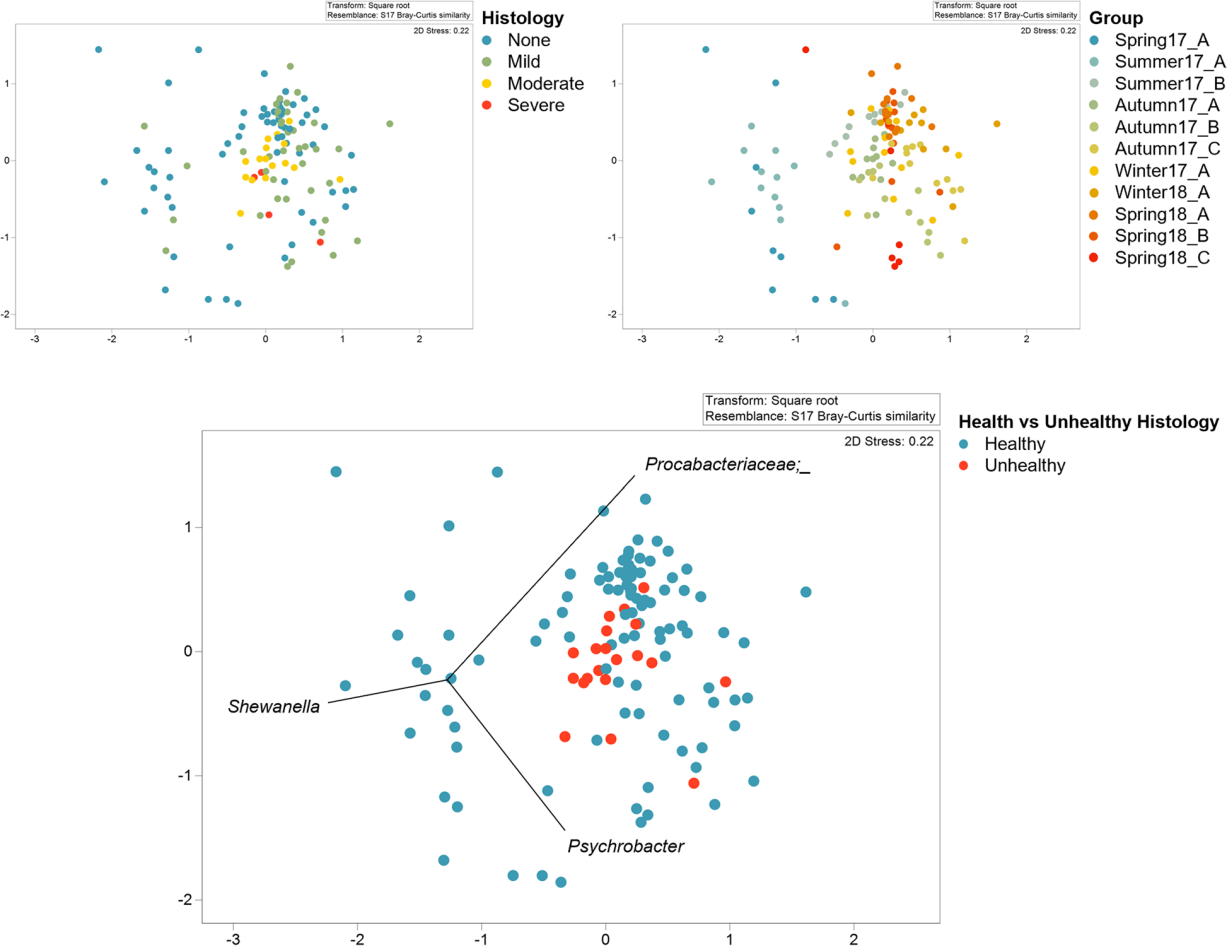
Non-metric Multi-Dimensional Scaling plot (nMDS) based on Bray–Curtis analysis (total = 128 fish). Coordinates 1 (x axis) and 2 (y axis) are plotted. Identical plots were colored to reflect the results of histology scoring (upper left), sampling group (upper right), and gill health (lower) to allow observation of ordination of samples based on these classifiers. Plotted taxa with a Pearsons rank correlation > 0.56 are *Shewanella*, *Psychrobacter*, and *Procabacteriaceae* (unassigned genus). These taxa apply to all plots, but are labelled on the gill health ordination only

Following taxa assignment of Amplicon Sequence Variants (ASVs) and filtering of non-bacterial derived sequences an average of 4275 sequences were obtained per sample (with four samples removed from the analysis due to overall read counts that fell below the rarefation threshold). A total of 627 bacterial ASVs were obtained, representing a total of 29 phyla, 83 classes, 145 orders, 265 families, and 499 genera (Table S4). Sequencing data have been made available via the NCBI database under accession number PRJNA667072. Alpha and beta diversity of the population were assessed using in-built features of R and the Primer 7 program. Trends in alpha diversity were apparent across the sampling period, with variation both in evenness, species diversity, and taxonomic richness throughout the annual production cycle. However, when fish were grouped by severity of histopathology, less variation was apparent (Figure S3). Non-metric Multi-Dimensional Scaling (nMDS) for ordination of Bray–Curtis similarity values (Fig. 4) was considered more appropriate for assessment of beta diversity than principal component analysis (PCA) (Figure S4) due to the non-continuous nature of feature count data. Fish gill community composition appears variable between individuals in the nMDS plot, with sampling visit timing appearing to influence observable dissimilatory (Fig. 4). Variation in microbial community composition between concurrently collected individuals (within sampling groups) is observable, as is some dissimilarity between temporally related sampling efforts, with concurrently collected samples appearing to ordinate in closer association than those collected at different times. Greater similarity between immediately sampling groups presents a picture of gradual community change over time and relatively greater dissimilarity between groups that were not sequentially sampled. Community composition of the first and second visits (Spring17_A in May 2017 and Summer17_A in June 2017) appear most divergent from subsequent visits, with these samples ordinated further from others in nMDS. Visualization of class-level taxonomy of all identified isolates suggests that while some taxa are variably present across the gills of individuals during the project duration, many taxa are consistently present, albeit altered in abundance. It is this variation in abundance rather than variable presence/absence of relatively abundant taxa that appears to contribute to dissimilarity between samples (Figure S7).

While much of the dissimilarity between individual samples appears explainable by sampling visit timing, ordination of samples by histology score and overall gill health does suggest an additional influence of gill pathology on community structure. Taxa with a Pearson rank correlation > 0.56 are labelled within the gill health nMDS plot (Fig. 4). These taxa were *Shewanella*, *Psychrobacter*, and an unassigned *Procabacteriaceae* isolate.

### Factors associated with altered microbial structure

#### Environmental and temporal influences on microbial beta diversity

On-farm data regarding daily temperature and other environmental parameters were not available for this study, however nearby weather station data (primarily from monitoring at the nearby town of Oban) are available and are provided in Tables S6a and S6b. During the time-period when samples were collected, this proxy data demonstrated predictable trends for the West Coast of Scotland, with high rainfall and altered air temperature in line with seasonal shifts. This information was accessed through the online historical weather platform ‘Visual Crossings’ [59]. After careful consideration, it was determined that the longitudinal field-based design of this study precluded modelling of the influence of these proxy environmental parameters on microbial communities due to potential divergence of proxy parameters from location-specific values and the lack of resolution within the study design, where extended periods occurred between sampling visits. Therefore, in lieu of assessing the influence of individual environmental parameters upon community structure, sample timing was considered a temporal influence on groupings. PERMANOVA analysis of square-root transformed data indicated that the variable of sampling group is a statistically significant predictor of dissimilarity between overall community composition of individual fish gills (pseudo-F = 3.684 *p* = 0.001). Subsequent ANOSIM testing then identified statistically significant differences between the community composition of individual sampling groups. Based on results of ANOSIM testing, most sampling groups were considered significantly different from some other groups. Spring17_A, Summer17_A, Autumn17_A, and Autumn17_B were consistently significantly divergent (*p* = < 0.01) from all other sampling groups (Table S5).

#### Gill health and microbial beta diversity

Additional variables explored via PERMANOVA analysis included the influence of histopathology on community structure. When fish were grouped as ‘none’, ‘mild’, ‘moderate’ or ‘severe’ based on composite histopathology scoring, PERMANOVA failed to find significant community-wide differences driven by total numerical histological scores (pseudo-F 1.878, *p* = 0.441). However, it was considered that this PERMANOVA analysis might be limited by the relatively small numbers of fish within the dataset characterized as having numerical scores within the ‘severe’ histological presentation (total = 3). To address this limitation, fish were grouped by histopathology score as having relatively healthy gills (none and mild pathology; total histology scores zero to six) and relatively unhealthy gills (moderate and severe pathology; total histology scores of seven and higher). Repeat PERMANOVA testing when fish were grouped in this manner found higher between-cluster variation (pseudo-F 2.398) with a significant *p*-value (*p* = 0.001).

#### Gill disease and specific microbial taxa

Classification of ASV’s to genus-level using the SILVA database demonstrates the presence of variable taxa throughout the sampling period. Use of SIMPER analysis identified and ordered taxa driving differences between gills with varied severity of pathology (Table 2). Based on this, a total of 7 unique taxa were determined to account for approximately 25% of dissimilarity between relatively healthy and relatively unhealthy gills: Candidatus *Branchiomonas*, *Rubritalea*, *Procabacteriaceae* (unassigned genus), *Shewanella*, *Psychrobacter*, Candidatus *Piscichlamydi*a, *Pseudoalteromonas*, and *Rhodobacteraceae* (unassigned genus). A list containing many of the same microbial genera was obtained when SIMPER analysis was applied to contrast the ten fish meeting the case definition of suffering from AGD against those that had no evidence of AGD (Table S7). Of the taxa identified through nMDS and SIMPER analysis, unpaired t-testing with Benjamini-Hochberg correction for multiple comparisons demonstrated significant differences in abundance of Candidatus *Branchiomonas* (*p* = 3.7e-08), *Rubritalea* (*p* = 0.005) and *Procabacteriaceae* (*p* = 0.039) between healthy and unhealthy gill health states. Use of a similar approach to explore any association of AGD with the microbial community identified a single ASV assigned as *Flavobacterium* as significantly varied between AGD positive fish and those that did not meet the case definition of AGD (*p* = 0.005). Results of unpaired t-testing of taxa identified by SIMPER and nMDS analysis are presented in supplementary materials (Figures S5 and S6).

**Table 2:** SIMPER analysis to identify microbiota contributing to dissimilarity between healthy and unhealthy gill health states

Genus	Average abundance relatively healthy gills (histology scores 0-6)	Average abundance relatively unhealthy gills (histology scores >6)	Average dissimilarity	Dissimilarity/Standard Deviation (SD)	Percentage contribution to variance	Percentage cumulative contribution to variance
Candidatus *Branchiomonas*	0.85	1.97	3.07	1.36	5.15	5.15
*Rubritalea*	0.76	1.57	2.36	1.27	3.95	9.1
*Procabacteriaceae* (unassigned genus)	2.13	2.38	2.02	1.09	3.38	12.48
*Shewanella*	0.76	0.8	1.91	1.17	3.2	15.69
*Psychrobacter*	0.99	1.01	1.63	1.39	2.74	18.43
Candidatus *Piscichlamydia*	0.69	0.47	1.58	1.03	2.65	21.07
*Pseudoalteromonas*	0.68	0.42	1.44	0.97	2.41	23.49
*Rhodobacteraceae* (unassigned genus)	0.6	0.32	1.22	1.05	2.05	25.54
*Pseudomonas*	0.53	0.31	1.11	0.99	1.86	27.4
*Acinetobacter*	0.42	0.57	1.08	1.25	1.81	29.21
*Flavobacterium*	0.4	0.4	1.04	1.02	1.74	30.95
*Serratia*	0.92	0.82	0.98	1.2	1.64	32.59
*Tenacibaculum*	0.37	0.27	0.96	0.75	1.6	34.19
*Flavobacteriaceae* (unassigned genus)	0.34	0.35	0.95	0.96	1.59	35.78
*Chryseobacterium*	0.35	0.35	0.88	1.07	1.48	37.26
Candidatus *Fritschea*	0.11	0.36	0.76	0.95	1.28	38.54
*Sphingomonas*	0.31	0.18	0.73	0.89	1.23	39.77
*Vibrio*	0.22	0.25	0.72	0.8	1.21	40.98
*Arcobacter*	0.26	0.19	0.66	0.84	1.11	42.09
*Sphingomonadaceae* (unassigned genus)	0.27	0.18	0.64	0.85	1.07	43.16
*Herbaspirillum*	0.19	0.12	0.56	0.65	0.93	44.09
*Hymenobacter*	0.12	0.26	0.54	0.79	0.9	44.99
*Colwellia*	0.18	0.08	0.45	0.54	0.76	45.75
*Mesorhizobium*	0.1	0.14	0.43	0.63	0.72	46.48
*Loktanella*	0.15	0.13	0.43	0.54	0.72	47.19
*Aeromonas*	0.04	0.19	0.42	0.63	0.71	47.9
*Stenotrophomonas*	0.17	0.1	0.42	0.67	0.7	48.6
*Delftia*	0.09	0.13	0.39	0.59	0.66	49.26
Candidatus *Nomurabacteria* (unassigned class, order, family and genus)	0.19	0.02	0.39	0.56	0.66	49.91
Candidatus *Finniella*	0.15	0.07	0.38	0.55	0.64	50.55

Gills were subclassified as ‘healthy’ (with low pathology scores, 0 - 6) and ‘unhealthy’ (high pathology scores, >7) to group fish based on histology scores obtained through the quantitative scoring approach described above. The microbial dataset was then was interrogated using SIMPER analysis (Primer-7). SIMPER analysis identified the most variable taxa between these groups. Identified taxa are presented in order of greatest percentage contribution to dissimilarity between groups

## Discussion

### Microbial communities with temporal and environmental factors

Results demonstrate a clear temporal trend in altered gill microbial community structure throughout the study period, with both PERMANOVA and ANOSIM analysis indicating that overall community composition was strongly influenced by sampling group (timing of sampling) (Table S5). This community change between sampling groups was potentially influenced by varied factors, including environmental parameters, on-farm activities, time at sea, as well as progressive microbial growth and random colonization and extinction of microbial isolates. ANOSIM results demonstrate significant differences between community structure at most of the eleven sampling time points, although interestingly not all. For example, Spring18_A and Spring18_B were not significantly different in community composition, and neither were Winter17_A and Winter18_A, despite being collected two months apart (Table S5). These findings suggest microbial community may have been relatively more stable in colder months and more dynamic during summer and autumn. Alternatively, findings could be interpreted to mean that the community gradually becomes more stable after adaptation to the marine environment, or that lower disease challenge and relatively few on-farm activities that might drive community change in colder months result in a less labile community during this time. Further study will be required to understand seasonal differences, but these results present novel insight regarding the gill bacterial community structure of farmed fish throughout the year.

Previous publications have noted seasonal and environmental drivers of variation in gill bacterial communities [60, 61]. Environmental parameters likely present an important driver of the temporally associated community change observed in this study. Given that environmental conditions were uncontrolled throughout the study period, fish were exposed to the combined influence of multiple environmental factors known to be drivers of community restructuring, including varied temperature, salinity, and pH. Previous research regarding the external microbiota of chum salmon (*Oncorhynchus keta)* demonstrates that a temperature change from 13 °C and 8 °C (an alteration of 5 °C) can induce significant alteration to the surface microbial community [62]. Temperature also influences the microbiota of Atlantic salmon, with variation in water temperature from 10.1 to 18.5 °C (an alteration of 8.4 °C) altering the fecal microbiome [63]. Although water temperature data for the specific sampling site were not available, the maximum recorded air temperature within the proxy dataset was 24 °C, and the minimum was -3 °C (Table S5b). Given this fluctuation, and given that sea temperature is influenced by air temperature [64], water temperature was a likely contributory variable in environmentally driven community change in this dataset. Varied pH and salinity are additional known drivers of microbial community restructuring in fishes including salmonids [16, 17]. Although we do not have pH or salinity data for the sampling site, the marine cage where the study population was maintained was located within a tidal sea loch where salinity will have been altered by tidal patterns as well as rainwater run-off following precipitation. Rainfall data were not available for the sampling site specifically, but regional weather data used as a proxy shows variation in average daily rainfall in the region from 50.4 mm to 0 mm (Table S5b). Therefore, whilst we cannot link salinity or pH directly to altered community structure in this study, influence of salinity is strongly suggested as an influence particularly upon samples collected at the start of the sampling period (groups Spring17_A and Summer17_A), given the recent transfer of those fish from freshwater to a marine setting. Sampling was initiated shortly after the freshwater to marine transition of the population, and thus it was considered that adaptation to altered salinity was a likely factor in observed dissimilarity between these initially collected groups and subsequently sampled groups in the nMDS plots (Fig. 4).

Being within the open water of the marine environment, additional environmental influences on the gill microbial community also cannot be excluded. Harmful environmental organisms like phytoplankton and jellyfish, both associated with damage to gill tissues for altered fish health, [65, 66] were not kept from entering net pens. In addition to causing physical damage to gill tissue such as was observed via histopathological assessment of gills in this study, jellyfish are documented hosts of potential fish pathogens within their own microbiome and are considered a possible route of vector transmission of bacterial organisms to fish [67, 68]. Microalgae, cyanobacteria, diatoms, and dinoflagellates may also potentially influence the gill microbial community. Microalgae *Chlorella pyrenoidosa* has been demonstrated to impact diversity, composition, and co-occurrence patterns of the gill-associated bacterial communities [69], while the cell structure and toxins from other harmful algae can negatively impact host health [70–72]. Influence of on-farm activities such as husbandry practices applied to the entire population potentially also influenced community structure in this study. Events that involved fish handling or treatments during this year-long study included introduction of lumpfish between the second and third sampling visits (Summer17_A and Summer17_B), as well as introduction of ballan wrasse between the fourth and fifth sampling visits (Autumn17_A and Autumn17_B). Treatments with hydrogen peroxide and warm water were applied for control of sea lice between visits six and seven (Autumn17_C and Winter17_A) as well as visits nine and ten (Spring18_A and Spring18_B) respectively. Previous publications demonstrate that both chemical and antibiotic treatments can alter the external microbial communities of fish [25, 73]. Similarly, any on-farm activities that caused stress to fish potentially altered or depressed host immune functions [74], and therefore might also have influenced community restructuring. Co-habitation of other fish species has the potential to have introduced novel microbial taxa that may have been harmful to the study population [75]. These on-farm treatments and husbandry events therefore potentially also acted as important factors that influenced the dynamic gill microbiome of all fish during the sampling period. Future research in a controlled environment might explore the specific alterations observed following application of individual husbandry practices through more frequent sampling of the microbial community.

In addition to environmental (external) drivers, host factors and temporal stochastic processes are well recognized influences upon microbial community change [76, 77] that might also have impacted this dataset. Although fish were of the same genetic stock, the age and size of fish varied during this study (Table S1). These host-associated parameters might be considered temporal factors, although they were altered in parallel with progression of sampling, making their influence challenging to parse from the influence of mixed external factors such as environmental drivers of microbial restructuring. With animals being maintained as a single population residing in a shared environment, the influence of environmental factors was theoretically shared. However, despite progressive temporal factors altering in tandem across the sampled population and the theoretically shared influence of host variables such as genetics, notable individual variation in the microbial consortia was still apparent between fish sampled concurrently (Fig. 4). Therefore, additional factors in driving individual variation must be considered. Samples were collected from gill tissues, a mucosal surface of fish with well documented immunological function [78, 79]. Thus, the influence of exclusively neutral influences on the microbial community of sampled gills seemed improbable, although random colonization and microbial loss likely did contribute in some capacity to observed variation [80]. Given the presence of gill pathology within the population, and the well documented association of gill disease and microbial organisms, gill pathology was considered a strong candidate variable towards explaining within-group community variation.

### Microbial communities and gill health

As an immunologically active organ and site of various commercially important diseases in Atlantic salmon [81, 82], gills represent an organ for which an understanding of harmful impact of microbial growth and scenarios that represent an unhealthy microbial community are of importance to understand. Immunological cells and their products are hypothesized to apply selective pressure to resident microbiota of fish mucosal surfaces, however, in times of stress or colonization by pathogens, these functions may be altered, resulting in an altered or ‘dysbiotic’ microbial community [83]. Microbial alterations that precede or follow disease events as well as microbial components that might limit growth or success of pathogenic isolates therefore present priority areas of research in the study of the Atlantic salmon microbiome. Towards enhanced understanding of the association between host and microbial interactions in the Atlantic salmon gill, we elected to investigate whether there existed a significant association between gill pathology and gill microbial structure in the study population.

Existing research highlights that genus-level community composition can be highly variable across individuals, where microbiota might have shared functional but significantly varied taxonomic composition [84]. In the wild, this variation might be explained by divergent life histories, diet, and genetic background [85]. However, in an aquaculture setting where these factors are controlled, various additional drivers of altered or disrupted community structure have been proposed, including influence of antimicrobial treatments or on-site activities such as net cleaning [86, 87]. Stress itself is understood to be an important host factor not only in predisposing microbial growth [88, 89], but also in explaining individual variation between the external epithelial communities of salmonids [90]. An obvious cause of stress in the population sampled as part of this research would be pathological tissue damage and disease in gills, stressors that can alter tissue integrity and disrupt immunological function [74].

Comparison of sampling groups in this study via ANOSIM testing identified that, in addition to between initially sampled groups (Spring17_A and Summer17_A), the microbial community of fish sampled during periods with highest gross gill damage and average histological scores (Autumn17_A and Autumn17_B) were consistently significantly different from most other sampling timepoints (Table S5). Although no significant association with microbial structure was seen when microscopic pathology was compared across fish considered to have none, mild, moderate and severe histologic change, when fish were grouped into those with relatively healthy gills (histopathology scores 0–6) or relatively unhealthy gills (histopathology scores of seven and above), PERMANOVA testing did identify a significant difference. This grouping of gills as relatively healthy or unhealthy is consistent with previous uses of the published scoring system, where minor pathology of gills was not considered clinically important [40]. Results therefore suggest a significant association of the microbial community with altered gill structure (pathology) in sampled fish. These community differences were associated with altered gill structure rather than any specific cause of gill pathology. For the majority of fish in this dataset, a specific cause of observed gill pathology was not determined. Given their presence in the marine environment and likely exposure to mixed infectious and non-infectious stressors, it is unlikely that gill damage observed in any fish was associated with a single cause or agent, and in some cases, damage may have not been due to infectious agents. Few infectious organisms were directly observed within gill histological sections, although AGD was a potential infectious driver of altered gill health in a proportion of the sampled population. Presence of clinical AGD was confirmed in a total of ten individuals throughout the 13-month sampling period. Although additional fish may have tested positive for the agent *Neoparamoeba perurans*, had molecular testing been performed only these fish were confirmed to be suffering gill damage associated with amoeboid organisms through histopathology. Given the uncontrolled nature of the marine aquaculture setting, it is not possible to separate the influences of individual pathogenic conditions and non-infectious drivers of gill damage. However, use of the semi-quantitative scoring system allowed for comparisons across varied states of gill damage regardless of cause. Many of the pathological changes observed within gill tissues of fish in this study were non-specific in their association with any discrete diagnosis [91]. This study can therefore confirm an association of altered microbial communities with altered gill structure generally, rather than any specific cause of gill disease.

#### Association of altered gill health with specific microbial taxa

A great deal of previous research details the biodiversity of microflora in the marine environment. Some of the microbiota identified in this study are commonly reported components of marine microflora, such as *Flavobacterium*, and taxa such as *Vibrio* and *Pseudomonas* [92–95]. Other identified taxa, including Candidatus *Branchiomonas* and C. *Piscichlamydia,* are considered obligate intracellular bacteria of fish [96, 97], with little evidence of their presence out-with a host. It is understood that the gill microbiome of fish is distinct from surrounding water, potentially due in part to the different micro-environment for microbial growth, as well as host immunological factors [98–100]. However, the environment is the most likely source of gill microbiota, and differentiating those resident to gill tissue from environmental taxa as contaminants of sampled microbial communities is challenging. A limitation of this study is the lack of robust environmental sampling to contrast marine microbes to identified gill adherent microbiota. This means a proportion of reported variation between sampling groups and gill health states may be as a result of environmental microbial change over time or in different growth conditions. Microbes that are variably present or absent from the population seem likely candidates as contaminant organisms.

##### Taxa associated with unhealthy gills

A number of specific taxa were identified as variably abundant in fish of different gill health states. Analysis of microbial community components at ASV-level using SIMPER analysis identified the most variable taxa between gill health states (Table 2). Some of these bacteria had been previously associated with gill disease in Atlantic salmon, including Candidatus *Branchiomonas* and Candidatus *Piscichlamydia*, ASV’s that accounted for 5.15% and 2.65% of observed variation respectively (Table 2). There currently exists a lack of consensus on the role Candidatus *Branchiomonas* and Candidatus *Piscichlamydia* play in gill disease of Atlantic salmon. A modeling approach by Downes et al*.* found no consistent association of Candidatus *Branchiomonas* with altered gill histology [101], while Gunnarsson and colleagues concluded that Candidatus *Branchiomonas* is at times correlated with gill disease in Atlantic salmon, and Gjessing et al*.* suggest Candidatus *B. cysticola* is a major contributor to CGD in Atlantic salmon [102, 103]. CGD is a disease in Atlantic salmon noted as involving multiple infectious agents including Salmon gill poxvirus [104, 105], but Candidatus *Branchiomonas* and Candidatus *Piscichlamydia* are also considered primary infectious agents as part of the gill condition epitheliocystis [106]. Only two fish in this study were noted to have evidence of epitheliocystis-type lesions (cystic structures containing granular, basophilic material within a thin eosinophilic capsule in the lamellae of gill tissue), and no fish from this study were considered to have histological changes in keeping with the presentations of Salmon gill poxvirus or CGD [54]. Despite this, Candidatus *Branchiomonas* was present with higher relative abundance in more damaged gills within this study**.** However, results demonstrate that this bacterium was also present on the surface of healthy gills, albeit in lower abundance. This is in keeping with previous research which identifies Candidatus *Branchiomonas* as an abundant component of the rainbow trout (*Oncorhynchus mykiss*) gill microbiome when no gill lesions were reported [107]. Based on our findings, altered abundance of these microbes rather than their presence or absence within the gill microbiome appears linked to varied gill health outcomes. While there remains much to be understood regarding the specific circumstances in which Candidatus *Branchiomonas* might act in gill pathologies, and whether presence of these microbes is a driver of gill pathology or as a consequence, these findings suggest the role of this microbe appears to be as part of a community associated with gill disease, rather than as a primary pathogen. These results therefore provide enhanced information regarding what specific microbes might indicate a gill ‘pathobiome’; an unhealthy or disadvantageous microbiome [13], and evidence of microbes for which the relative abundance might represent informative indicators of Atlantic salmon population health.

Abundance of the taxa *Rubritalea* was also significantly correlated with gill damage (Figure S5). Interestingly, our findings are in contrast to previous research in European Seabass (*Dicentrarchus labrax*), where *Rubritalea* was found to be the dominant ASV in the mucus microbiota of fish and present in higher abundance in uninfected fish relative to those infected with *Vibrio harveyi* [108]. The role of *Rubritalea* in gill health outcomes is therefore at present unclear, however, these results suggest that at least in this population of Atlantic salmon, increased relative abundance of this microbe may have been associated with negatively altered gill health.

##### Taxa associated with healthy gills

In addition to the taxa associated with gill damage, some ASVs identified in this study were considered to have significant negative associations with gill pathology. These included an ASV classified to genus-level as *Shewanella,* and another classified only to family-level as *Procabacteriaceae*. *Shewanella* abundance was identified in nMDS ordination and SIMPER analysis as a factor in observed community dissimilarity (Fig. 4), however average abundance did vary noticeably between individual fish (Table 2). *Shewanella* has been demonstrated to be an abundant component of the gill microbiota in other studies of Atlantic salmon gills across diverse geographical locations [109], suggesting a potential role for this microbe as a universal microbe of Atlantic salmon gills. Additionally, *Shewanella* abundance has already been proposed as an important factor in the relationship of the Atlantic salmon gill microbiome and gill disease [34]. *Shewanella* may therefore represent an important core component of the Atlantic salmon gill microbiome and bioindicator of Atlantic salmon gill health. In contrast, *Procabacteriaceae* represents a bacterial family with only a single documented genus (*P. procabacter*) for which relatively little information is available beyond being an obligate symbiont of acanthamoeba [110, 111]. To our knowledge, and aside from in related work by these authors studying the same population [38], this work represents the first description of *Procabacteriaceae* on Atlantic salmon gills. The documented hosts of *Procabacteriaceae* are *Acanthamoeba*, a genus of amoebic organisms occasionally identified as infectious agents in fish but that can also be noted without apparent disease [112]. Interestingly, recent research examining the intracellular bacteria of *Neoparamoeba perurans* (causative agent of AGD), found that *Vibrio* are a predominant bacterial genus [113]. Identification of *Procabacteriaceae* may therefore indicate presence of less harmful amoebic organisms on the gills of fish within this study. However, given the methodology of microbial sampling via gill biopsy rather than gill swabbing, the *Procabacteriaceae* observed may also represent a novel intracellular microbe of Atlantic salmon gill cells themselves. If identified *Procabacteriaceae* were located within gill cells, this ASV may be disproportionately represented within this dataset due to our sampling methodology. Similarly, failure of other studies to identify the microbe may be due to the use of swabbing methodology. Based on these results, we unfortunately cannot say what relationship the identified *Procabacteriaceae* had to gill tissues in fish, and whether it is a true component of the fish microbiome or an intracellular microbe of associated acanthamoeba. However, regardless of localization, the taxa identified as *Procabacteriaceae* appear benign, and a potential indicator of gill health within this population of farmed fish. Any future studies that identify this family of bacteria might consider imaging the ultrastructure of gills to understand niche partitioning of this microbe, perhaps with a similar study design to previous research of Candidatus *Branchiomonas* [103].

##### Taxa associated with specific disease conditions

Through histopathology, in addition to non-specific gill changes, some individuals within the population were observed to have patterns of gill change and pathogen presence consistent with specific infectious diseases. Two fish had low prevalence of histological changes characteristic of epitheliocystis, and a total of ten fish were also considered to be infected by AGD based on the histological case definition of presence of amoebic organisms with a parasome and hyperplastic gill changes [37, 55–58]. Microbes identified within the dataset that are known to be associated with infectious gill disease included Candidatus *Branchiomonas* and Candidatus *Piscichlamydia*, as well as the genera *Tenacibaculum* and *Winogradskyella*. The genus *Tenacibaculum* is known to contain multiple species pathogenic to fish including *Tenacibaculum maritimum*, causative agent of Tenacibaculosis, and both *Tenacibaculum* and *Winogradskyella* have been previously associated with AGD [114, 115]*. Winogradskyella* was present at low relative abundance in sampling groups Autumn17_A, Autumn17_B, and a single fish in Autumn17_C, sampling groups when gill pathology was at its most severe (Fig. 3). ASV’s identified as Tenacibaculum were relatively common within the dataset (Table S4). Although *Tenacibaculum dicentrarchi* has been previously associated with gill lesions from AGD [114], the genus *Tenacibaculum* contains many species. Given the sequencing approach employed in this study, where only a portion of 16S rRNA material was amplified and sequenced, our results cannot classify identified *Tenacibaculum* to species level. It is possible that multiple *Tenacibaculum* species were present within the dataset and although SIMPER analysis identifies this genus as partly accounting for dissimilarity between gill disease states (Table 2), it was not identified to be significantly differently abundant between gill health states. ANOVA testing indicated that only taxa within the genus *Flavobacterium* were significantly differentially abundant between AGD positive and AGD negative fish, with relatively lower abundance in fish with clinical AGD. However, few fish as part of the population overall were considered to have clinical AGD, and *Flavobacterium* were not significantly varied between general gill health states. Previous research identifies species of *Flavobacteria* as apparently benign components of the gill microbiota [32], although there are species that are pathogenic to Atlantic salmon, causing conditions such as cold water disease [116]. Overall, the apparent significant association of *Flavobacterium* with AGD in this study should be interpreted cautiously, particularly given the uncontrolled environmental conditions and likely mixed influences of infectious and non-infectious gill disease in conjunction with AGD. Future probiotic research that aims to identify beneficial microbes might explore the utility of *Flavobacterium* in the context of AGD in Atlantic salmon. In this research however, results cannot support an association of microbial taxa with any discrete cause of gill damage due to the uncontrolled study environment. Instead, results associate differences in relative abundance of specific microbial taxa only in association with general pathology, providing insight regarding general indicators of gill health.

### Gill health monitoring and trends in epidemiology of disease

Many previous studies have employed similar gross gill scoring systems to ours in assessing severity of both generalized gill damage and AGD [117, 118]. Regarding assessments of gill change in this study histological and gross scoring indices were largely in agreement: Low incidence of gross pathology during initial sampling visits in spring 2017 was mirrored by low histological scores, and greatest incidence of gross change coinciding with elevated histology scores. Although ten fish were observed to suffer histological change without scoring of clinical pathology (Table S2), the histology in these fish was not scored above ‘mild’, and so this disparity was likely due to the microscopic nature of many structural changes that can occur within gill tissues. An additional two fish that had gills where gross change was apparent were without notable microscopic change (with gill scores from 0-3 classified as ‘none’) (Table S2). These findings highlights the selective nature of histopathology, a technique that provides highly specific assessment of tissue integrity but for which appraisal of only a small section area of an organ is possible. Use of histological scoring systems and gross assessments in combination such as employed here therefore seems an effective combined approach to quantify gill damage.

Use of the gross and histological scoring systems in this study provided quantitative assessment of changes to gill structure during the sampling period. This fish health monitoring demonstrated a seasonal trend in gill disease, with peaks of both macroscopic (Fig. 1) and microscopic (Fig. 2) pathology in late summer and early autumn**.** This is a trend well recognized by aquaculture producers of marine cage Atlantic salmon. Research emphasizes that the occurrence of seasonal gill disease is not incidental, but rather a complex annual emergence influenced by varied epidemiological drivers including host, environment, and pathogenic factors [4, 119]. These drivers of disease emergence and severity are often interrelated for the combined outcome of impaired animal health [120]. In the case of Atlantic salmon within the marine environment, these are factors such as increased water temperature, fish stress, and pathogen proliferation. Based on regional temperature data (Table S4), fish in this study likely encountered elevated water temperatures during late summer into early autumn. Warmer waters can be stressful to fish, and they can also benefit growth of environmental organisms that can cause gill damage, including harmful phytoplankton, Cnidaria, and pathogenic microbes [4, 119]. Critically, an accumulating body of research suggests that in addition to the epidemiological triad of host, environment, and pathogenic factors that drive gill disease, the microbiome itself may also be important in determining health outcomes. Many publications now document an association of fish disease and the resident microbiota [121–123]. Therefore, although much is still to be learnt regarding the cause and effect of this association, this publication adds to the building evidence of an association of resident microbial communities and gill health.

#### Dysbiosis

The term dysbiosis is widely used to describe divergence from a more diverse microbiome community structure to one with fewer ASVs. Many studies also link dysbiosis with negative impacts for fish health [23, 34]. However, in the case of the gill microbiota of the Atlantic salmon studied here, the microbial communities associated with diseased gills were not observed to be notably reduced in richness, evenness, and diversity relative to fish with healthier gills. When sampling visits were compared, periods of lower community diversity were detected, but when fish were grouped by severity of gill pathology this reduced community diversity was not noticeably associated with gill disease (Figure S3). Lack of an observable trend in microbial diversity or richness with gill damage in this study presents an interesting finding in that it suggests that altered microbial community richness and diversity were not directly linked to any alterations in gill health in these fish. The significant influence of sample timing on microbial community structure with inferred influence of factors such as environmental parameters on community composition does not therefore appear to be a negative restructuring. Negative microbial community restructuring in this study instead takes the form of altered abundance of specific taxa such as Candidatius *Branchiomonas.*

Previous studies have noted that following disruption, the microbial consortia of fish skin will not return to its previous community composition, even with removal of the factor that precipitated change [124]. These previous observations are mirrored here, where the gill community appears dynamic throughout the thirteen-month sampling period. These findings present an interesting question as to what best represents a healthy gill community, and how best to characterize dysbiosis in different scenarios, given the variable nature of stochastic colonization or extinction of microbial taxa [20]. For example, microbial community structure changed dramatically in the first weeks of the study following introduction of the population to seawater. The microbial community structure of the first and second sampling visits (Spring17_A and Summer17_A) are notably divergent from subsequent samples (Fig. 4). Previous studies have noted similar fluctuations in the microbiome of skin and gills during this period of transition and over time in recirculating systems [16, 125, 126]. The process of smoltification and transition from freshwater to the marine environmental is stressful for fish, and disease outbreaks do occur. However, these results show that although following transfer to the marine environment the microbial community of gills is significantly and rapidly altered, there was in this instance no directly observable consequence to gill health. A limitation of this research may have been concurrent sampling for gill damage and microbial community, for failure to capture subsequent community change with worsening or healing gill pathology. Similarly, the relative infrequency of sampling may have failed to capture transient pathology or gill damage subsequent to unmeasured events such as jellyfish blooms or net cleaning. However, instead of negative change associated with on-farm events or environmental change, restructuring of the gill microbial community identified in this study may represent a benign or even adaptive restructuring. Many factors will have influenced community change, including presence of existing taxa that preclude or encourage growth of others [127, 128], new random colonization’s, and modulation of the host immune function due to stress and altered salinity [129, 130]. Future studies might more closely assess gill microbial community structure during the saltwater adaptation of juvenile salmon alongside monitoring for gill disease to identify any periods of true dysbiosis and recovery with associated negative impacts on host health. Overall, although the sampling design of this study lacked the fine resolution to identify gradual microbial community change, and so fails to establish causative relationships between gill damage and microbial community change, results still present an interesting picture of annual community change, and taxa of altered relative abundance during periods of gill damage.

## Conclusions

Relatively lower ASV richness and diversity such as are described during dysbiosis were not linked here with incidence of gill damage. Therefore, instead of describing observed community change as dysbiosis, we concluded that results support continual restructuring of the community structure of marine cage farmed Atlantic salmon gills, likely driven by mixed influence from environmental factors, random change, or even selective host-driven adaptation. However, some changes to microbial community structure were significantly correlated with occurrence of gill pathology in this study, with a number of specific ASVs identified as importantly contributing to dissimilarity across gill health states. These identified taxa might make meaningful indicators of general gill health and potentially of use to producers seeking to monitor mixed gill disease in marine net pens, or as targets of future research regarding manipulation of the Atlantic salmon gill microbiome. Altered abundance but maintained presence of identified taxa suggests the existence of a ‘core’ community throughout the sampling period, and it is altered abundance of a proportion of taxa within the core community that might present useful indicators of gill health. Relative abundances of microbes such as Candidatus *Branchiomonas* and *Procabacteriaceae* associated in this study with altered gill health states might be useful in the future as microbial indicators of gill health in instances where material for histopathology cannot be obtained, when a less invasive sampling approach is sought, or when repeated sampling for health monitoring from individuals is desired. Regarding probiotic development, whilst *Shewanella* is a microbe frequently described in gill tissue, *Procabacteriaceae* represents a novel biomarker of gill health. Further work will be required to understand in greater detail the relationship of *Procabacteriaceae* with gill health, particularly its location on or within gill tissues, and whether *Procabacteriaceae* represents a universal component of the gill microbiota, or one unique to this population. Important to remember is that these results represents only a proportion of the overall population, and although findings were potentially mirrored in other net pens also suffering gill pathology, only a single net pen was examined. Each population of fish experiences different stressors during their production cycle, including environmental challenges, handling stress, and specific disease challenges. Therefore, it is unclear at present whether *Procabacteriaceae* might be a universal marker of gill health or specific to this population. Future studies including tank-based challenges might consider manipulating identified microbial taxa on the gills of fish explore the influence of different microbial taxa on Atlantic salmon gill health more directly. Experimental study designs that examine the impact of on-farm activities such as ballan wrasse introduction on microbial community structure would further these research goals, providing insight regarding microbial community change and any positive or negative gill health consequences following specific events.

Overall, much is still to be learnt regarding what constitutes a healthy or maladapted gill microbial community, and whether altered microbial communities are a symptom of or predisposing factor to gill diseases. Despite significant association of microbial community and gill pathology in this study, questions remain as to their causal relationship: Observed gill pathology and disease outcomes may have been precipitated by varied microbial structure, or varied microbial structure may have occurred because of altered gill environment and structure with diseases such as AGD. However, the results of this study do provide clear advancement to the field, with new insight into the microbial community structure of gills during an annual production cycle of Atlantic salmon.

### Supplementary Information

Additional file 1: Figure S1: Gross changes on fish gills.

Additional file 2: Figure S2: Rarefaction curves.

Additional file 3: Figure S3: Diversity, evenness, and richness throughout the sampling period.

Additional file 4: Figure S4: Principal Component Analysis and loading values.

Additional file 5: Figure S5: Relative abundance of significantly varied microbial taxa across different gill health states and sampling groups.

Additional file 6: Figure S6 Additional select microbial taxa across different health states and sampling groups.

Additional file 7: Figure S7: Class level relative abundance during sampling timeline.

Additional file 8: Table S1: Fieldwork data. Table S2a + S2b: Index and ancillary criteria. Table S3: Histology scores. Table S4: Relevant abundance of sequencing results classified to the lowest taxonomic level (genus where relevant). Table S5: ANOSIM results. Table S6a + S6b: Environmental proxy data. Table S7: SIMPER results from fish grouped by AGD status.

## Data Availability

The sequence dataset generated in this study are available via the NCBI database https://www.ncbi.nlm.nih.gov/genbank/ under accession number PRJNA667072.
