# Supplementary figures and images for: Association of microbial community structure with gill disease in marine-stage farmed Atlantic salmon (Salmo salar); a yearlong study
Source: BMC Vet Res. 2024 Aug 1;20:340. doi: 10.1186/s12917-024-04125-5 (PMC11293161; doi:10.1186/s12917-024-04125-5)

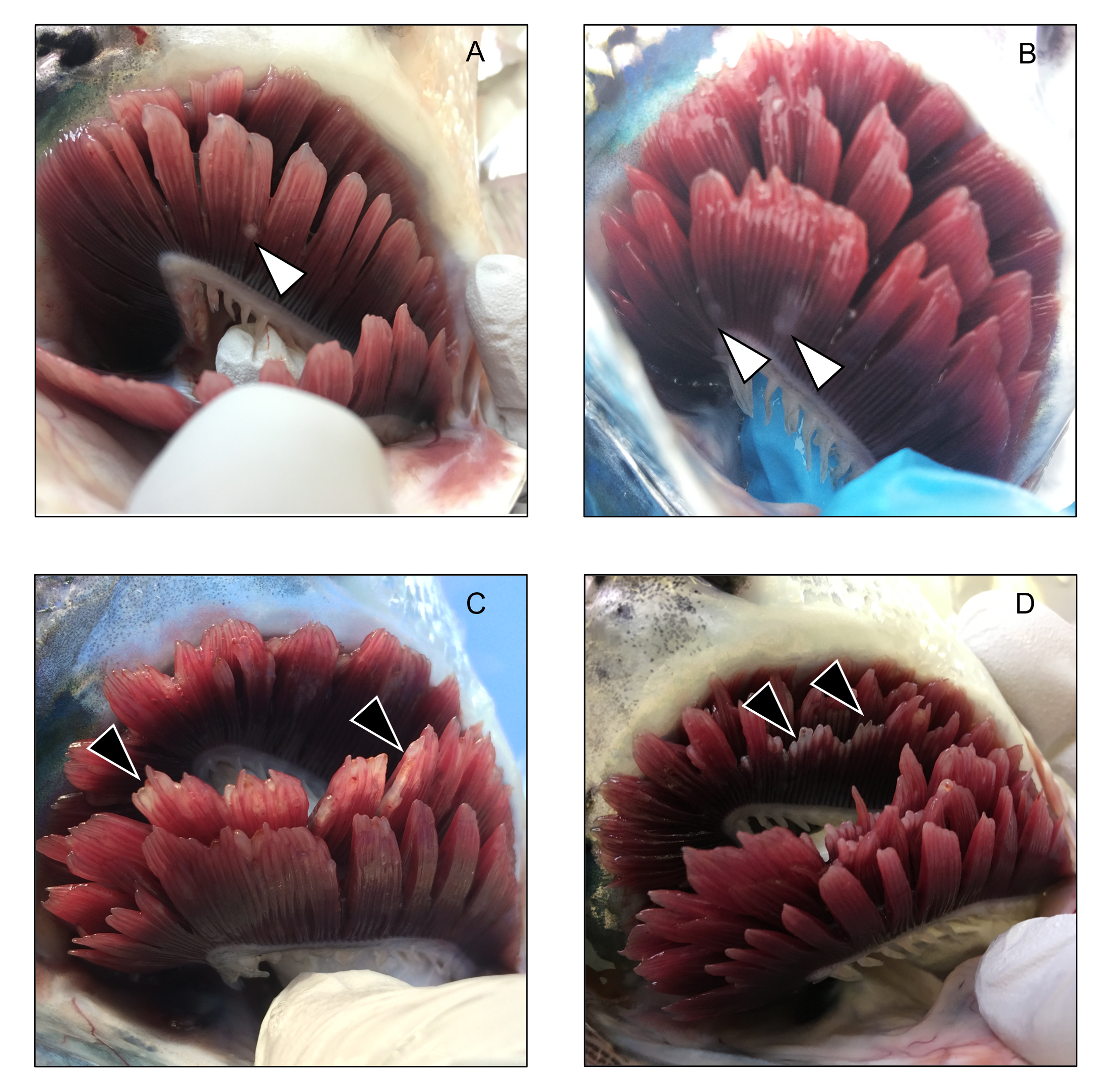

Supplement: Supplementary file 1 — Additional file 1: Figure S1: Gross changes on fish gills. [file 12917_2024_4125_MOESM1_ESM.tif]

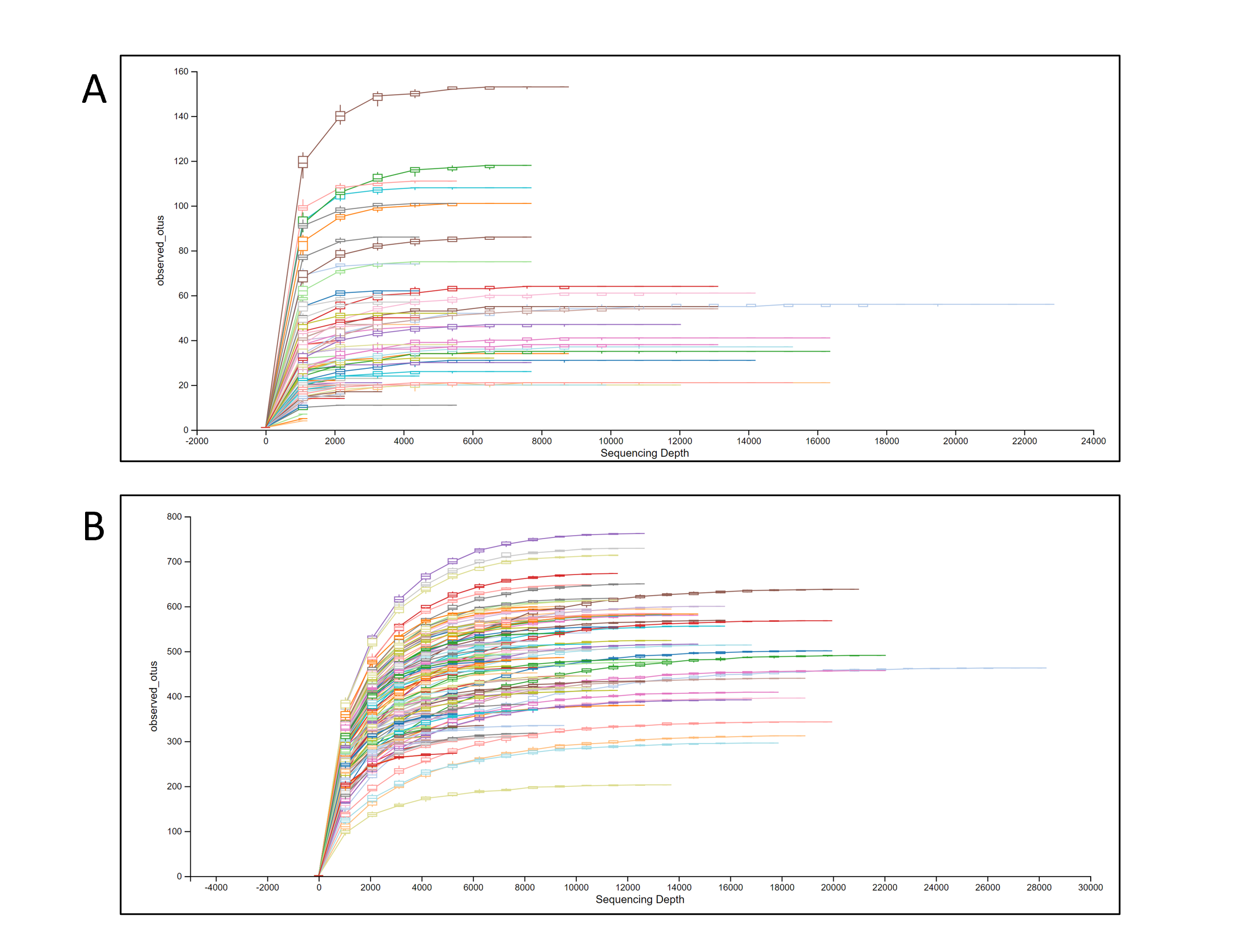

Supplement: Supplementary file 2 — Additional file 2: Figure S2: Rarefaction curves. [file 12917_2024_4125_MOESM2_ESM.tif]

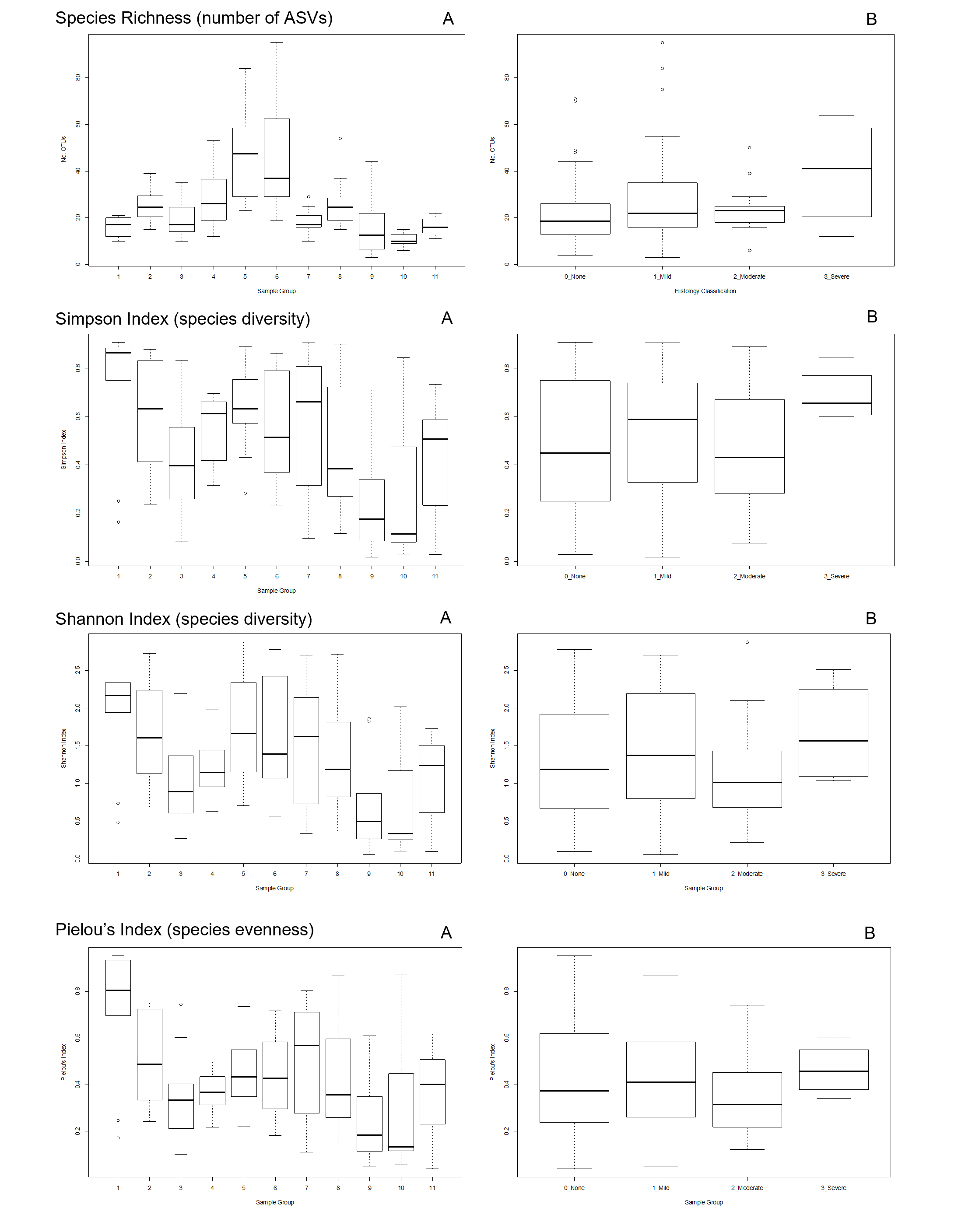

Supplement: Supplementary file 3 — Additional file 3: Figure S3: Diversity, evenness, and richness throughout the sampling period. [file 12917_2024_4125_MOESM3_ESM.tif]

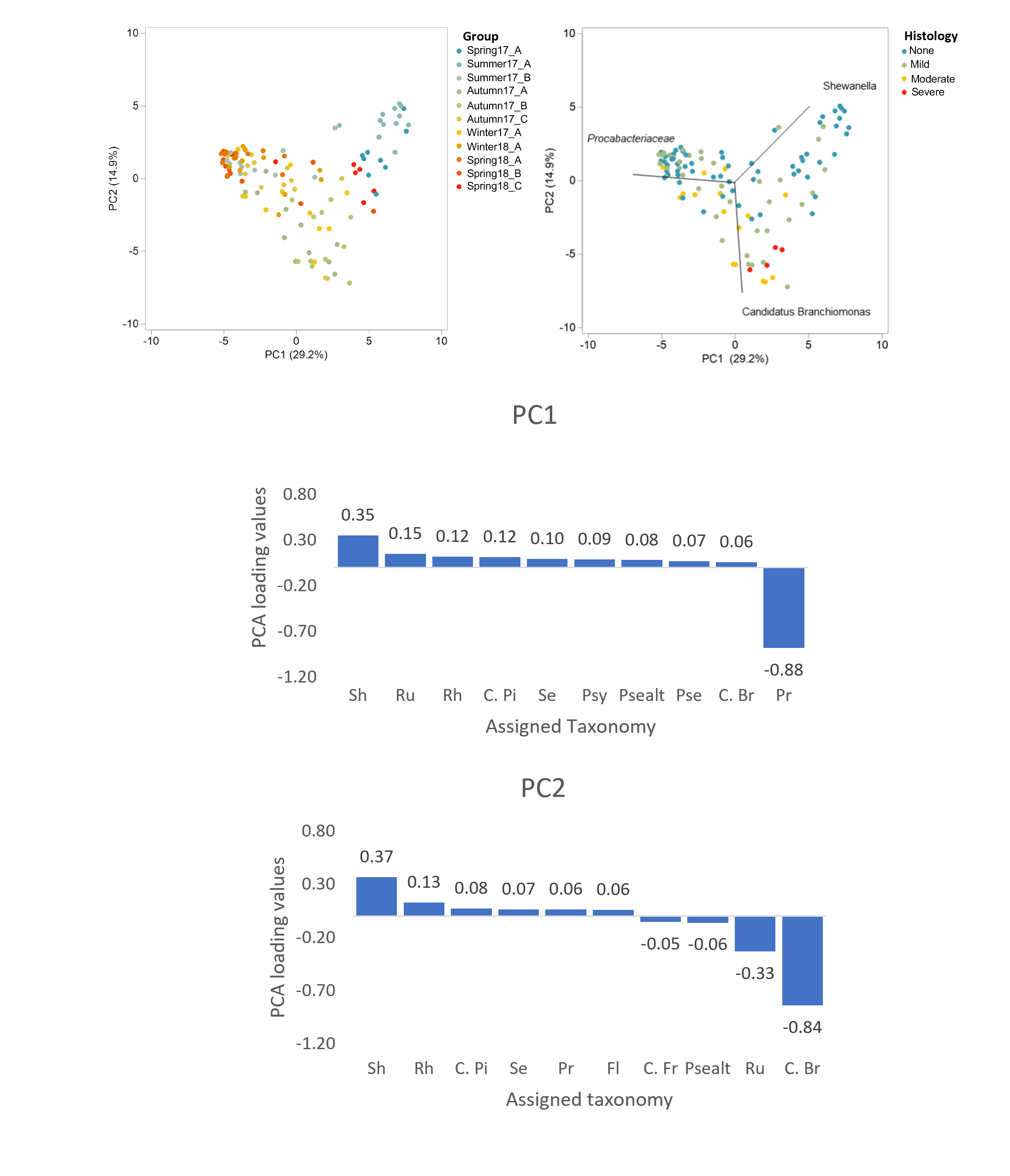

Supplement: Supplementary file 4 — Additional file 4: Figure S4: Principal Component Analysis and loading values. [file 12917_2024_4125_MOESM4_ESM.tif]

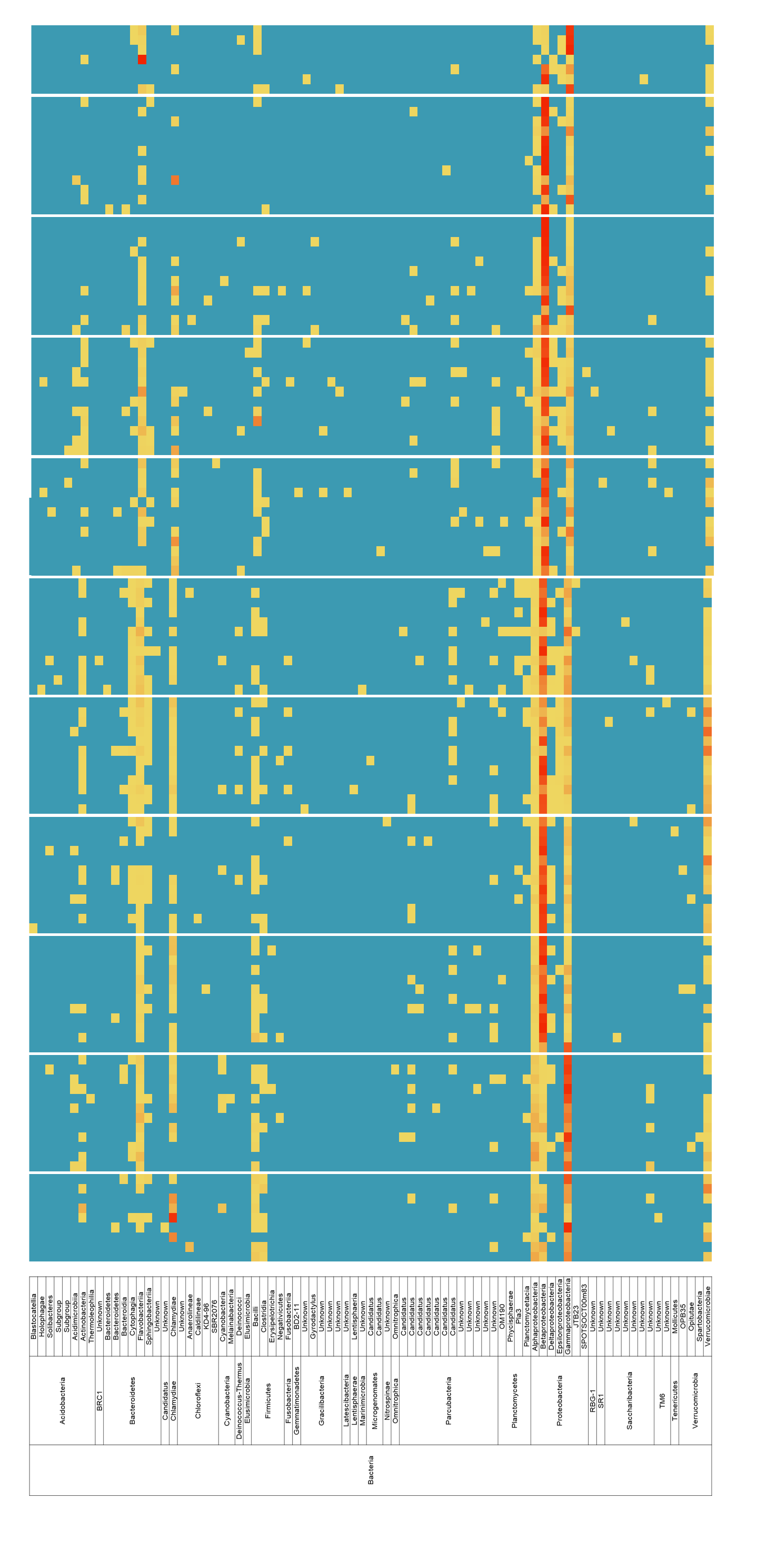

Supplement: Supplementary file 5 — Additional file 5: Figure S5: Relative abundance of significantly varied microbial taxa across different gill health states and sampling groups. [file 12917_2024_4125_MOESM5_ESM.tif]

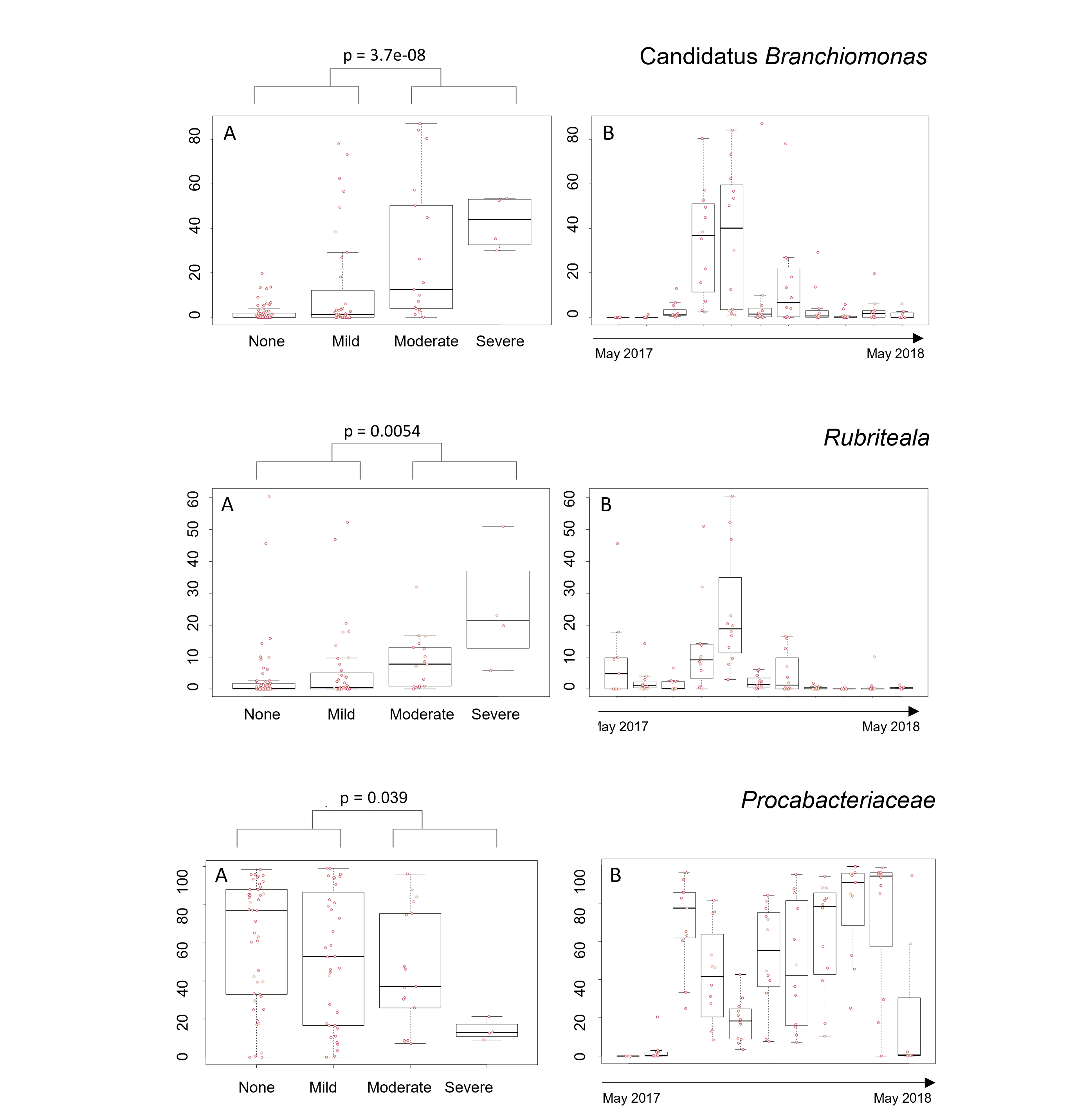

Supplement: Supplementary file 6 — Additional file 6: Figure S6 Additional select microbial taxa across different health states and sampling groups. [file 12917_2024_4125_MOESM6_ESM.tif]

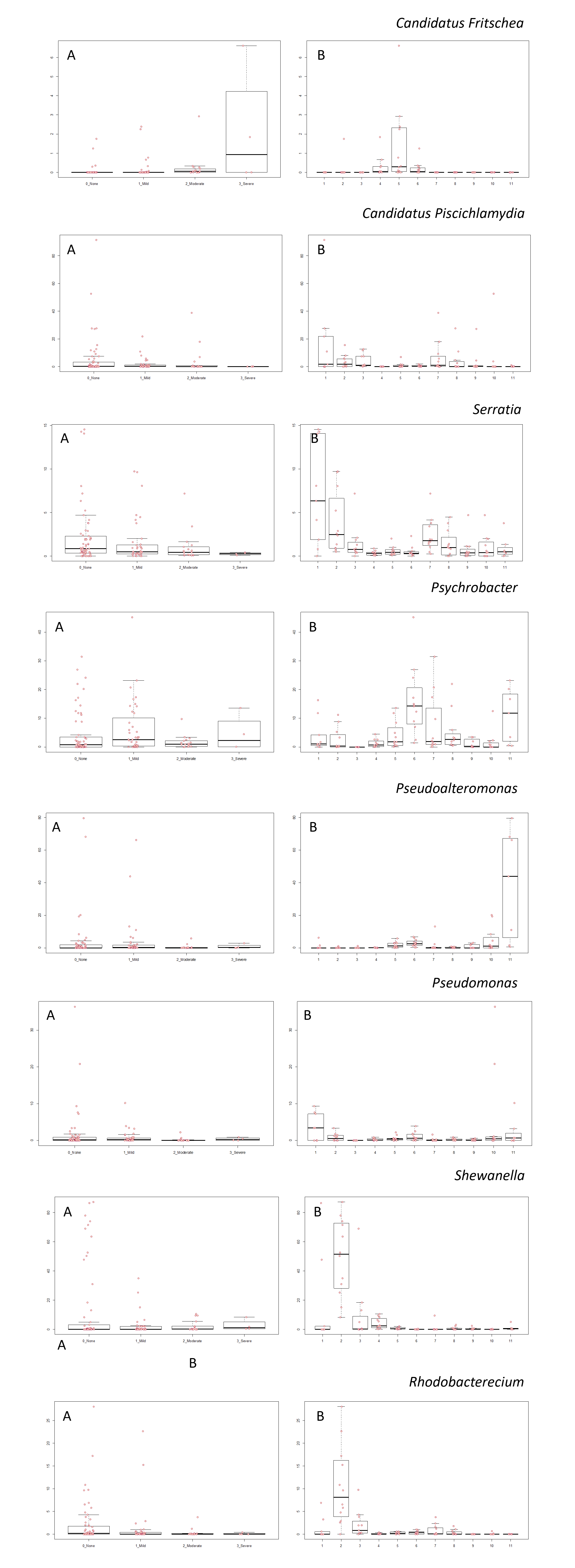

Supplement: Supplementary file 7 — Additional file 7: Figure S7: Class level relative abundance during sampling timeline. [file 12917_2024_4125_MOESM7_ESM.tif]
